# Factors Associated with Cognitive and Functional Performance in Indigenous Older Adults of Nariño, Colombia

**DOI:** 10.1155/2019/4542897

**Published:** 2019-10-01

**Authors:** Yenny Vicky Paredes-Arturo, Eunice Yarce-Pinzon, Diego Mauricio Diaz-Velasquez, Daniel Camilo Aguirre-Acevedo

**Affiliations:** ^1^Mariana University Faculty of Humanities and Social Sciences, Psychology Program, Pasto, Colombia; ^2^Mariana University Health Sciences Faculty, Occupational Therapy Program, Pasto, Colombia; ^3^The University of Edinburgh, Global Health Unit, Social Policy Programme, Edinburgh, UK; ^4^University of Antioquia, School of Medicine, Medellín, Colombia

## Abstract

**Introduction:**

Ethnicity in Latin America is a factor of poverty and social exclusion. Like in developed countries, demographic, medical, psychosocial, global cognitive, and functional variables interact in a complex relationship on the elderly population. Such interaction should be considered to determine cognitive and functional performance using screening tests. The aim of this study was to evaluate the demographic, medical, and psychosocial factors affecting global cognitive performance as well as functional activities.

**Methods:**

The study was conducted in a Colombian elderly indigenous population which included a sample of 518 adults. This research employed a structural model of latent factors to assess the effects of demographic, medical, and psychosocial factors on cognitive and functional performance. The model was estimated by least squares and used a maximum-likelihood procedure, and it was determined RMSEA, TLI, and CFI to assess the model's goodness of fit. The categorical variables used in the model were as follows: (1) demographics, (2) psychosocial factors, (3) medical condition, (4) global cognition, and (5) functional factors.

**Results:**

Demographics, in addition to medical and psychosocial factors, were related to global cognition and functional factors (RMSEA = 0.051, CI 90% 0.045–0.057, CFI = 0.901, and TLI = 0.881).

**Conclusion:**

These results provide strong evidence about the complex relationships among demographics, medical conditions, and psychosocial factors and their influence on global cognition and functional performance in Colombian indigenous elderly population.

## 1. Introduction

In Latin America, ethnicity is a strong risk factor associated with poverty, inequality, and social exclusion, mainly in rural areas and in low-income families. Ethnicity has a negative impact on the most vulnerable sectors of the population such as children and the elderly [1]. Ethnicity, as a condition of social inequality, when it is related to differences in language and low education, can impact global cognitive tracking evaluations. Such impacts can cause heterogeneity in the execution of tests, limiting an individual's operational characteristics despite a priori adjustments that could be proposed in [2].

On this subject, there are studies that inform about the direct and indirect effects demographic conditions, general health problems, and psychosocial variables have on the execution of cognitive tracking tests as well as functional evaluations. Such data can be adjusted into a multivariate model of complex interactions of 3 factors (the factors have been medical history, global mental state, and gender) related to the functional condition and cognitive involvement of the elderly [3]. The model shows a complex structure of interrelations between factors such as genetics, emotions, sociodemographic factors, and health status. The health status is usually associated with the comorbidity of chronic diseases. All these associated factors entail a greater risk for the development of neurodegenerative diseases with negative consequences in daily life activities [4]. In this manner, the epidemiological profile of the disease in the indigenous community has gradually changed from a scenario characterized by infectious and parasitic disorders to an increase in the rates of chronic noncommunicable diseases. Nevertheless, this study does not disregard the strong relationship between cardiovascular risk factors, functional performance, emotional state, cognitive impairment, and dementia in the population of indigenous older adults [5, 6].

These conditions are shared by indigenous communities in other Latin American countries, where the precarious health situation of these contexts constitutes another relevant factor for the increase in dependence and mortality [7]. The increase in risks such as chronic diseases, dependence, and mortality could be related to an acculturation process of the native population. The natives have acquired lifestyles which in part have increased their life expectancy but, on the other hand, increased the prevalence of chronic diseases, causing dependence on the elderly [7, 8]. The aim of this study was to evaluate the demographic, medical, and psychosocial factors affecting global cognitive performance and functional activities, through a structural model of latent factors. The model was applied to a group of indigenous senior citizens in the province of Nariño, Colombia. This study is of great relevance because the most important problems in the adult stage are the loss of cognitive and functional abilities, even more regarding ethnic communities belonging to a scenario of old age and multimorbidity [9]. Therefore, knowing the state of cognitive and functional performance of these adults allows to understand the levels of dependence of indigenous population from others to do activities, an important indicator of their health condition.

## 2. Materials and Methods

This project was designed as an observational and analytical study and is based on a transversal descriptive nature. The reference population was 5759 older indigenous people who belonged to 13 subregions of the former jurisdiction of Obando in the province of Nariño, in southern Colombia, data obtained by the DANE Census and its estimate for 2016. In this way, the sample size included a total of 518 older adults. To estimate the sample, a prevalence of 50% was considered, a margin of error between 3 and 5% with a 10% increase in potential losses. The sample size was considered appropriate for this study, taking into account the rule of 10 participants per variable in the structural model, for a ratio of 23 participants per variable (*n* = 518/22 variables in the model) [8]. The inclusion criteria were as follows: being an adult 60 years of age or older, belonging to an indigenous council, and voluntarily accepting participation in the study by signing the informed consent. The exclusion criteria were established based on the presence of some medical or cognitive implications that prevented the application of the protocol.

### 2.1. Valuations

We considered the following variables: sociodemographic, medical, nutritional, cognitive, and functional factors. These factors were evaluated with a multidimensional questionnaire previously validated [10] and a self-report provided by each participant or a relative responsible for caregiving. The demographic characteristics were evaluated depending on the particularity of these; age was approached in ranges since it allows a better analysis of it; in relation to the education variable, it was guided by categories that may or may not read and write and by primary, secondary, technical, undergraduate, and postgraduate levels; regarding income, it was investigated based on the current legal minimum wage. The medical component was consulted for the presence or not of medical illness. The nutritional assessment was determined using the full version of the Mini-Nutritional Test [11]. This evaluation was carried out by an interdisciplinary team in areas such as nursing, neuropsychology, physical therapy, and occupational therapy. The presence of malnutrition or risk of malnutrition was defined as follows: malnutrition was considered when a person scored in the Mini-Nutritional Test less than 17 points (<17); malnutrition risk relates to a score between 17 and 23.5; and good nutritional status relates to 24 points or more. The study applied the Mini-Mental State Examination (MMSE) test for cognitive evaluation. For this study, it was used the cutoff point of ≥24 [12].

Additionally, it was used the Rowland Universal Dementia Assessment (RUDAS) test considering a cutoff point of 21. The latter was used because it does not contain biases linked to instruction level. Its ordinal scoring system is 30 points [13]. Further, the study applied the subjective memory complaints (SMC) consisting of 15 questions to determine the functioning of the patient's memory in daily life using a Likert frequency scale. The maximum score for the Likert scale is 45 and the cutoff point is 19 [14]. Likewise, depressive symptoms were evaluated according to a self-report that used the Yesavage geriatric depression scale [15]. The scale considers three categories (normal, moderate depression, and severe depression) according to a total score obtained from a sum of 15 items. A score of 0 to 5 denotes normal (no depression), 6 to 10 moderate depression, and 11 to 15 severe depression. The social variables were evaluated through the Medical Outcomes Study—Social Support Survey (MOS-SSS) with 20 questions in total, related to the perception of support of the individual, in addition to exploring about the accompaniment in the instrumental, affective, social, and family dimensions. The response options are given through a Likert scale of 1 (never) to 5 (always) [16].

The study used the Lawton and Brody scale to assess the degree of functional independence regarding instrumental daily life activities [17]. The scale assesses the ability to perform tasks that involve the handling of habitual utensils and routine social activities. The Lawton and Brody scale uses 8 items for assessment (ability to use the phone, shopping, preparing food, taking care of home, laundry, use of transportation means, responsibility for medication, and economy administration) and assigns a numerical value 1 (independent) or 0 (dependent) for the assessment process. The final score results from the sum of the values in each answer and goes between 0 (maximum dependence) and 8 (total independence). Concerning the psychometric properties, the scale is reliable with a Pearson coefficient of interobserver reliability of 0.85 and good concurrent validity with other scales of cognitive and daily activities. It assesses the ability of a person to perform ten basic activities of daily life in a dependent or independent way. The Barthel index score ranges from 0 (completely dependent) to 100 (completely independent). A degree of dependence is established according to the score obtained, being the most frequent cutoff of 60 points (between moderate and mild dependence) and 40 points (between moderate and severe dependence) [18].

### 2.2. Ethical Issues

The study was reviewed and approved by the Bioethics Committee of Mariana University, located in Pasto, Nariño, Colombia. The study complies with the recommendations provided by the Resolution 8430 of 1993 of the Colombian Ministry of Health to conduct research. Additionally, the study fulfils the Helsinki Declaration of the World Medical Association guidelines [19]. Further, the investigation sought approval from each indigenous community council. This process was carried out in the first semester of 2017.

### 2.3. Statistical Analysis

**Figure 1:**
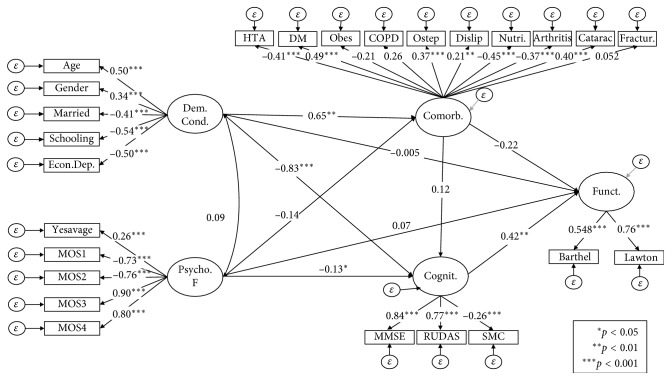
Structural model of the relationship between demographic conditions, comorbidity, social support, and its relationship with cognitive profile and functionality. Econ. Depend : economic dependency; MOS-SSS1 : informational/emotional dimension; MOS-SSS2 :instrumental dimension; MOS-SSS3 : positive social interaction dimension; MOS-SSS4 : affective support dimension; Demog Cond :demographic conditions; Psycho F : psychosocial factor; Comob : comorbidity; Cognit : cognition; Funct : functionality; HT : hypertension; DM : diabetes mellitus; Obes : obesity; COPD : chronic obstructive pulmonary disease; Osteop : osteoporosis; Dyslip: dyslipidemia; Nutr :nutritional status; SMC : subjective memory complaints.

The study used frequency to describe variables identified as demographic characteristics, medical history, social support, and cognitive performance. The study used the percentage value to describe categorical variables and mean along with standard deviation to describe continuous variables. According to these characteristics, we explored differences in the cognitive performance evaluated by the MMSE and RUDAS using the *t*-test for independent samples (gender, place of residence, economic dependence, and medical history) and analysis of variance (ANOVA) for level of schooling, marital status, and home income. MMSE had to be transformed using a cubic power since it did not fulfil the assumption of homogeneity in variance. Additionally, the Scheffe test was used when comparing groups as a post hoc test. The Pearson correlation coefficients between MMSE and RUDAS were calculated with the MOS-SSS dimension scores. It was proposed a structural model (Figure 1) assuming latent variables such as (1) demographics, (2) psychosocial factor, (3) health condition, (4) cognitive dimension, and (5) functional dimension. The model assumed that demographic factors and psychosocial factors influenced a person's health condition, in addition to the cognitive and functional dimensions. The RMSEA index (root mean square error of approximation), CFI (confirmatory fit index), and TLI (Tucker–Lewis index) were determined to assess the model's goodness of fit. A good adjustment was assumed if the RMSEA index was less than 0.08 and the CFI and TLI >0.90 [20]. Given that the model included categorical variables, we used a robust estimation method through weighted least squares with a likelihood ratio (WLSMV), using a diagonal weight matrix with standard errors and chi-square test statistic with an adjusted mean and variance [21]. The results in the model are presented as standardized coefficients that indicate the correlation between the factors and dimensions. The analyses were carried out in the SPSS IBM software version 23 and in MPLUS 7.0 [22].

## 3. Results

### 3.1. Description of Cognitive and Functional Performance according to Demographic Characteristics

From the 518 participants in the study, it was found an average in the MMSE of 22 points (SD = 5.4) and in the RUDAS scale of 20.3 (SD = 4.7). The SMC showed an average of 23.6 (SD = 9.9). Concerning the functional scales, the average was 97 (SD = 7) and 6 (SD = 2) for the Barthel and Lawton scales, respectively. After adjusting for age and educational level, the correlation between RUDAS and MMSE was 0.60 (*p* < 0.001).  Table 1 presents the behaviour of the MMSE, the RUDAS, and the subjective complaints of memory and functional scales, according to demographic characteristics.

The correlation with age was −0.33 (*p* < 0.001), −0.36 (*p* < 0.001), −0.20 (*p* < 0.001), and −0.30 (*p* < 0.001) for the MMSE, the RUDAS, Barthel, and Lawton, respectively. Significant differences were found according to gender (MMSE (*t* = 4.4; gl = 506.0; *p* < 0.001) and QM (*t* = −2.2; gl = 510; *p* < 0.028)); level of education (MMSE (*F* = 91.3; gl1 = 2; gl2 = 515; *p* < 0.001), RUDAS (*F* = 29.3; gl1 = 2; gl2 = 515; *p* < 0.001), Lawton (*F* = 7.8; gl1 = 2; g2 = 515; *p* < 0.001), and Barthel (*F* = 4.3; g1 = 2; g2 = 515; *p*=0.014)); marital status (MMSE (*F* = 14.6; g1 = 2; g2 = 515; *p* < 0.001), RUDAS (*F* = 7.3; gl1 = 2; gl2 = 515; *p*=0.001), and Barthel (*F* = 6.0; gl1 = 2; gl2 = 509; *p* < 0.003)); and economic dependence (MMSE (*t* = 4.4; gl = 516; *p* < 0.001), RUDAS (*t* = 2.4; gl = 516; *p*=0.015), Lawton (*t* = 3.7; gl = 423; *p* < 0.001), and Barthel (*t* = 2.7; gl = 383; *p*=0.007)). No significant differences were found regarding the area of residence and home income.

### 3.2. Description of Cognitive and Functional Performance according to Medical History

Table 2 presents the cognitive and functional performance according to the presence of medical history. Hypertension was associated with MMSE (*t* = 3.0, df = 499, *p*=0.003), RUDAS (*t* = 2.8, GL = 188, *p*=0.005), and Yesavage (*t* = −2.0, df = 199, *p*=0.047). Diabetes was associated with MMSE (*t* = 2.6, df = 496, *p*=0.009), RUDAS (*t* = 2.2, df = 496, *p*=0.027), and Barthel (*t* = 1.6, df = 23.6, *p*=0.004). The presence of osteoporosis was associated with the scale of memory complaints (*t* = −2.8, df = 481, *p*=0.006) and Barthel (*t* = 2.2, df = 481, *p*=0.029). The correlation between the MMSE, RUDAS, Lawton, and Barthel with Yesavage was −0.257 (*p* < 0.001), −0.310 (*p* < 0.001), −0.183 (*p* < 0.001), and −0.233 (*p* < 0.001), respectively.

### 3.3. Relationship between General Cognitive Performance with Memory Complaints, Depression, Functional Scales, and Social Support

Table 3 presents the Pearson correlation coefficients between the MMSE and RUDAS with SMC, depression, functional scales, and social support. An inversely proportional correlation was observed in both scales regarding the scores related to subjective complaints of memory and depression. Additionally, a positive correlation was observed with the functional scales. Only the RUDAS showed significant correlation with MOS-SSS in its 4 dimensions and for the total scale.

### 3.4. Structural Model to Evaluate the Relationship between Demographic Conditions, Comorbidity, Social Support, and Its Relationship with Cognitive Profile and Functionality

Figure 1 presents the structural model that represents the relationship between demographic conditions, comorbidity, social support, and its relationship with cognitive profile and functionality. This model showed an adequate fit with an RMSEA index = 0.062 (CI 90% 0.057–0.067, CFI = 0.64, and TLI = 0.59). This model explains 34% of the variability of functionality, 59.8% of the variability of cognitive profile, and 44.9% of the variability of comorbidity. According to the modification indexes, the model improved the adjustment considering ([Supplementary-material supplementary-material-1]-Supplement) covariances between demographic condition (latent) with obesity and Yesavage; comorbility (latent) with Yesavage; cognition (latent) with Yesavage nutrition level, education, sex, economic dependency, and obesity; and functionality (latent) with Yesavage age and nutrition level. This last model showed an adequate adjustment with an RMSEA index = 0.042 (CI 90% 0.036– 0.047, CFI = 0.85, and TLI = 0.82). This model explains 36.0% of the variability of functionality, 63.0% of the variability of cognitive profile, and 39.9% of the variability of comorbidity.

## 4. Discussion

Nowadays, the relevance of research in ethnic minorities has received more recognition. The scientific community has begun to consider ethnicity as a potential differentiating factor in the aging process. This increased interest in this subject could be explained by the disparity in terms of health and illness compared to other contexts [23]. Furthermore, it could be explained by the need to better understand the interaction of health determinants' characteristics with this type of population given their ability to influence morbidity and mortality [24]. Thus, the aim of this study was to evaluate the demographic, medical, and psychosocial factors that affect global cognitive performance and functional activities in a group of indigenous older adults, using a multifactorial structural model of latent factors. The results of this study show a significant relevance of the aforementioned factors in cognitive and functional performance in the study group, evaluated through the multidimensional geriatric model.

Regarding the demographic factors, and specifically considering the education variable, it was evaluated based on the following categories: the person's ability to read and write or the lack of such skills and the level of instruction in primary school. In the evaluated subjects, a minimum proportion of them reached primary education and illiteracy prevailed significantly. This situation is corroborated not only in indigenous contexts but also in rural communities, where poor infrastructure conditions, difficult geography, dispersed population, and armed conflict have led to educational vulnerability [25]. Thus, in Colombia, the percentage of illiterate people is 1.9%, while among indigenous people that percentage amounts to 3.7%, or approximately 30,000 individuals [26]. In relation to gender, a higher prevalence of the male sex is observed. This is a datum that differs from the common theoretical references, especially in rural and urban contexts. Usually, there is more convergence towards a greater incidence of the female gender, something also referred to as the “feminization phenomenon” of aging [27].

In relation to marital status, the data show a significant percentage of older adults who belong to the married category. About this, it could be argued that marriage becomes a protective factor for these people because it determines an individual's main support network. Thus, with a partner, an individual's perception of support in matters related to emotional and economic stability improves [28]. Concerning income, it was determined that most of the subjects evaluated do not receive income. This reality provides an important account about the difficult economic situation in which indigenous elderly adults live. This problem is exacerbated if one considers the precarious health and the deep social inequalities they face [29]. This issue has also been observed in older adult farmers where demographic determinants are precarious [30]. Therefore, the demographic profile of this population is characterized by situations of extreme vulnerability around health conditions, education, infrastructure, and basic services (e.g., water and health care).

At the level of cognitive performance, this was determined through the application of the Mini-Mental and RUDAS scales cognitive. The latter was used for its psychometric properties which decrease schooling bias towards the subjects to whom it is applied given that most test items do not require basic instruction. Similarly, a lower cutoff point was chosen compared to other population groups, like from the rural or urban context. Despite this, the studied population obtained a significantly lower average score than expected. This situation is corroborated in a report obtained in studying an indigenous population of Putumayo, Colombia [31], where 87 % of the elderly presented mild cognitive impairment. Similarly, this type of data has been reported in more general rural contexts [27]. The above is argued based on the demographic characteristics of the population, where age represents a fundamental determinant in this type of performance in ethnic groups, whose gradual increase tends to decrease the ability to solve problems and process new information [32].

Likewise, gender is a significant factor in terms of cognitive functioning [33]. However, bibliographic references are still divergent on this issue. Traditionally, the existence of a differentiated profile is cognitively significant and can affect at cultural and functional levels [28]. Moreover, education is a variable that greatly affects cognitive performance. Low schooling could explain the substantial variation in cognitive test scores among different population groups [24]. In this way, the consensus is generally established that performance on low-educated individuals can result in two standard deviations below the normal average on the cognitive follow-up scales [33]. Indeed, such a situation was presented in the evaluated population where a percentage of them had basic primary education and in the worst case belonged to the illiterate category. Possibly, this situation can be explained due to schooling processes not consistent with their ethnic characteristics and cultural patterns, among others.

Further, in indigenous contexts, there is an increased risk of medical comorbidity characterized by the incidence of cardiovascular diseases and greater exposure to cardiometabolic risk factors, such as obesity, diabetes, and hypertension [34]. This situation would explain the health disparity of ethnic groups. In this sense, diabetes is a complex and socially mediated disease, explained by a series of factors that include colonization, a situation that has influenced the change in their healthy lifestyles [35]. Diabetes in the indigenous population is related to the prevalence and incidence of hypertension; hence, they have a bidirectional tendency. In this sense, high blood pressure represents a health risk and the main cause of disability in cognitive functioning. In previous research, hypertension was practically nonexistent among indigenous adults; however, the prevalence currently ranges from 29.7% of the before-mentioned population [36]. Several studies have referred to the epidemiological evidence regarding the relationship between diabetes and hypertension with cognitive functioning [37]. This is explained by the presence of numerous pathophysiological mechanisms that enhance cognitive impairment, which is related to impaired insulin function and vascular problems, all related to the beta-amyloid protein. Additionally, hypertension is a risk factor that generates lesions at the vascular level and can also cause ischemic lesions at the cerebral level [38].

Considering the emotional component, there was a higher frequency of depressive symptomatology in the indigenous elderly. This prevalence is also corroborated in other studies analyzing regular and indigenous elders in a rural context with a reported incidence of 80% of the population [30, 31]. Its diagnosis is important as it represents a criterion of fragility. It is also the main cause of emotional suffering and low quality of life at old ages [39]. In relation to the population evaluated, a significant percentage of them live in disadvantaged social situations. This precarious condition is related to unfavorable socioeconomic standards in which they have developed their lives, contributing to the prevalence of emotional disorders [31]. The previous argument is relevant if one considers the relationship between cognitive performance and cerebral reserve—the incidence of this latter variable increases the risk of mental deterioration. At the emotional component level, a study refers significant correlation between depressive symptoms and cerebral reserve (rho = 0.583; *p* < 0.001) among the indigenous community [31]. Therefore, it could be concluded that the presence of depressive symptomatology in the indigenous older adults evaluated could be related by the precariousness of demographic and psychosocial factors in which they are immersed. It is also clear that the armed conflict and colonization of these ethnic groups can have an important effect [29].

In another context, Early and colleagues argue that functional capacity is conditioned by multiple factors. In this sense, one of the most referenced variables in this type of research is gender. Apparently, women are more vulnerable to functional dependence attributed to their greater longevity in relation to men [5]. However, the results of the present investigation indicate otherwise. The above could be explained due to the agricultural work culture that women have carried out and was an aspect evaluated in the instrument. Depending on age, this variable seems to have a “dose-response association” in which, at an older age, there is a greater risk of functional dependence. This argument is evidenced in longitudinal and cross-sectional studies, in which a greater age range is perhaps the most important risk factor in the impairment of functional capacity [40]. Similarly, there is no continuous relationship between the increase in disability and age but tends to accelerate especially in states of greater longevity [41]. Furthermore, structural characteristics such as improvement in psychosocial and socioeconomic conditions could generate improvements in cognitive performance among elderly to prevent situations of functional dependence through the maintenance of healthy lifestyles. [5]. Besides, the functionality of older adults is associated not only with the performance of physical activity and social participation, but also with the social support they have, especially from the family, which otherwise may lead to deterioration for adequately coping with life, affecting their well-being and increasing functional dependence [42]. Thus, having strong family and social ties allows an emotional exchange that leads to a structural and functional strengthening of this group.

Equally, the functionality in elder adults would be related to the presence of depressive symptomatology, specifically, their inability to perform activities of daily life, determined by the complexity and time required to undertake tasks [43]. The related literature on this issue suggests that the dopaminergic hypoactivation process constitutes in the justification for the previous argument since a motivational process is necessary to carry out activities and sequence motor actions. Meanwhile, in a population of indigenous older adults, emotional participation can have a greater negative impact on functional capacity than chronic diseases [44]. Further, with respect to the cognitive component, the literature refers the fact that deficits in the higher mental processes determine participation and performance in daily life activities, affecting the quality of life in the elderly [45]. In this study, the results converge towards the presence of cognitive impairment that compromises functional capacity. However, some authors express that in ethnic groups without cognitive impairment, functional dependence can occur. The consensus on this issue indicates that in older adults, there is a certain commitment to perform instrumental activities, although there may be deficits in the performance of basic activities in advanced stages of dementia [46].

Referring to the strengths of the study, it could be argued that they are mainly related to the multidimensional evaluation performed for a specific type of population, that is, indigenous older adults. In addition, it is the first study of this nature conducted in Colombia. Therefore, these findings could guide the design of strategies, intervention programs, and structuring of public policies that could improve cognitive and functional performance among the population studied. Parallel to this, the main limitation of the investigation was its transversal nature. Also, some situations in the elderly are fluctuating, especially those related to the cognitive and emotional state.

## 5. Conclusion

In a group of older indigenous adults, conditions of extreme demographic vulnerability prevail, mainly in the field of educational training. Likewise, depending on the medical comorbidity, there is evidence of the presence of hypertension, diabetes, and depressive symptoms among the adults. This profile affects and predisposes the indigenous elderly to clinical conditions such as cognitive impairment and dementia.

## Figures and Tables

**Table 1:** Cognitive and functional performance according to the demographic characteristics of 518 indigenous senior citizens in the province of Nariño, Colombia.

Variables	Frequency	%	RUDAS		MMSE		SMC		Lawton and Brody		Barthel	
			Mean	SD	Mean	SD	Mean	SD	Mean	SD	Mean	SD
*Gender*												
Female	231	44.6	20.2	5	23.2	5	22.6	10	6	2	97	7
Male	287	55.6	20.4	4	21.2	5.4	24.5	9.7	6	2	96	7

*Age*	70.6	6.8										
60–65	130	25.1	22.4	4	24.5	4.3	22.5	9.5	7	1	98	5
66–70	149	28.8	20.7	4	22.3	5	23.1	10	6	2	97	6
71–75	111	21.4	20.3	5	21.8	5.4	25.1	9.8	6	2	96	8
76–80	78	15.1	18.4	5	20.7	5	24.4	10	6	2	96	6
80 or more	50	9.7	16.6	5	18.2	6.1	23.6	11	5	2	93	10

*Speaks native language*												
Yes	74	14.3	20.6	5	22.4	5.3	22.8	9.1	6	2	95	9
No	444	85.7	20.2	5	22.1	5.4	23.8	10	6	2	97	7

*Schooling*												
S/he cannot read/write	208	40.2	18.5	5	18.8	5.1	23.9	10	6	2	96	9
S/he can read/write	249	48.1	21.6	5	24.5	4.2	23.5	9.5	6	2	97	6
Basic primary education	61	11.7	21.2	4	23.4	4.4	23.4	11	6	2	98	6

*Residence*												
Rural	475	91.7	20.2	5	22.1	5.4	23.4	9.9	6	2	97	7
Urban	42	8.3	20.8	5	22.3	5.3	26.2	9.9	6	2	97	5

*Income*												
Without income	276	53.3	19.7	5	20.7	5.6	23.3	10	6	2	95	8
Less to a LMMW^*∗*^	237	45.8	20.9	5	23.6	4.6	24.2	9.6	6	1	98	8
Between 1 and 3 LMMW^*∗*^	5	0.9	25.2	4	28.6	1.5	15.8	15	7	1	99	2

*Marital status*												
Single	82	15.8	20.2	4	21	5.8	25.6	9.4	6	2	97	5
Married/consensual union	329	63.5	20.8	4	23.1	4.8	23	10	6	2	97	6
Widowed/divorced	107	20.6	18.8	6	19.9	5.7	24	9.9	6	2	95	10

*Economic dependency*												
Yes	356	67.6	19.9	4	21.4	5.4	23.8	9.9	6	2	96	7
No	162	32.4	21	4	23.6	4.9	23.4	9.9	7	1	98	6

^*∗*^LMMW: legal minimum monthly wage in Colombia.

**Table 2:** Cognitive and functional performance according to medical records of 518 indigenous senior citizens from the province of Nariño, Colombia.

Illnesses		Total, *n* = 518		RUDAS		MMSE		SMC		Lawton		Barthel	
		Frequency	%	Mean	SD	Mean	SD	Mean	SD	Mean	SD	Mean	SD
HT^*∗*^	Yes	128	24.7	19.3	5.4	21	5.4	24.3	10	6	2	96	9
	No	390	75.3	20.6	4.4	22.5	5.3	23.4	9.8	6	2	97	7

CVD^*∗∗*^	Yes	5	3.5	23	3.6	24.6	5	20	9.3	8	1	97	4
	No	513	96.5	20.3	3.6	22.1	5.4	23.7	9.9	6	2	97	7

Diabetes	Yes	24	4.6	18.4	5.9	19.5^*∗*^	6.2	27.2	9.1	5	3	93	12
	No	494	95.4	20.4	4.6	22.2	5.3	23.4	9.9	6	2	97	7

Dyslipidemia	Yes	99	19.1	20.2	5.2	22.3	5.7	24.9	9.6	6	2	96	7
	No	419	80.9	20.3	4.6	22.0	5.3	23.3	10.0	6	2	97	7

Obesity	Yes	30	5.8	20.4	4.3	23.5	4.5	23.4	12	7	2	96	6
	No	488	94.2	20.3	4.7	22.0	5.4	23.5	9.8	6	2	97	7

COPD^*∗∗∗*^	Yes	18	3.5	19.1	5.5	21.4	4.9	26.4	8.5	6	2	94	10
	No	500	96.5	20.3	4.7	22.1	5.4	23.5	10.0	6	2	97	7

Osteoporosis	Yes	53	10.2	20	4.9	21.6	5.2	27	9.9	6	2	95	9
	No	465	89.8	20.3	4.7	22.2	5.4	23.2	9.9	6	2	97	7

Arthritis	Yes	247	47.7	20.1	4.5	21.8	5.2	24.7	9.9	6	2	96	7
	No	271	52.3	20.5	4.9	22.5	5.4	22.7	9.8	6	2	97	7

Cataracts	Yes	120	23.2	20	5.2	20.8	5.5	25.1	10	6	2	96	7
	No	398	76.8	20.7	4.5	22.5	5.2	23.2	9.8	6	2	97	7

Fractures	Yes	89	17.2	20.1	4.8	22.6	5.1	24.4	9.1	6	2	96	8
	No	429	82.8	20.3	4.7	22.0	5.4	23.5	10.1	6	2	97	7

*Yesavage*													
Normal		181	34.9	22	4.1	23.8	4.8	18.5	10	6	2	98	5
Moderate depression		252	48.6	19.7	4.5	21.4	5.6	25.2	8.8	6	2	97	6
Severe depression		85	16.4	18.4	4.3	20.4	5.4	30	6.8	6	2	93	11

*Nutritional status*													
Normal		382	73.7	20.6	4.7	22.5	5	23.2	10	6	2	97	6
Malnutrition risk		136	26.3	19.4	4.7	21	6.1	24.8	9.6	6	2	91	10

^*∗*^HT: hypertension; ^*∗∗*^CVD: cardiovascular disease; ^*∗∗∗*^COPD : chronic obstructive pulmonary disease.

**Table 3:** Pearson correlation coefficients between MMSE and RUDAS with the MOS-SSS and its dimensions.

	MMSE	RUDAS
Subjective memory complaints (SMC)	−0.178^*∗∗*^	−0.250^*∗∗*^
Yesavage	−0.244^*∗∗*^	−0.297^*∗∗*^
Barthel	0.249^*∗∗*^	0.232^*∗∗*^
Lawton and Brody	0.385^*∗∗*^	0.347^*∗∗*^
Social support (MOS-SSS)		
Informational/emotional dimension	0.052	0.107^*∗*^
Instrumental dimension	0.077	0.145^*∗∗*^
Positive social interaction dimension	0.065	0.159^*∗∗*^
Affective support dimension	0.031	0.140^*∗∗*^
Total (MOS-SSS)	0.064	0.150^*∗∗*^

^*∗*^<0.05, ^*∗∗*^<0.01.

## Data Availability

The university in which we work does not allow us to share the database.

## Supplementary Materials

Supplementary Materials Structural model was used to evaluate the relationship between demographic conditions, comorbidity, social support, and its relationship with cognitive profile and functionality. This model showed an adequate adjustment with an RMSEA index = 0.042 (CI 90% 0.036— 0.047, CFI = 0.85, and TLI = 0.82). This model explains 36.0% of the variability of functionality, 63.0% of the variability of cognitive profile, and 39.9% of the variability of comorbidity.
