# Supplementary material for: Factors Associated with Cognitive and Functional Performance in Indigenous Older Adults of Nariño, Colombia
Source: J Aging Res. 2019 Oct 1;2019:4542897. doi: 10.1155/2019/4542897 (PMC6791244; doi:10.1155/2019/4542897)
Supplement: Supplementary Materials — Structural model was used to evaluate the relationship between demographic conditions, comorbidity, social support, and its relationship with cognitive profile and functionality. This model showed an adequate adjustment with an RMSEA index = 0.042 (CI 90% 0.036— 0.047, CFI = 0.85, and TLI = 0.82). This model explains 36.0% of the variability of functionality, 63.0% of the variability of cognitive profile, and 39.9% of the variability of comorbidity. [file 4542897.f1.pptx]

## Slide 1
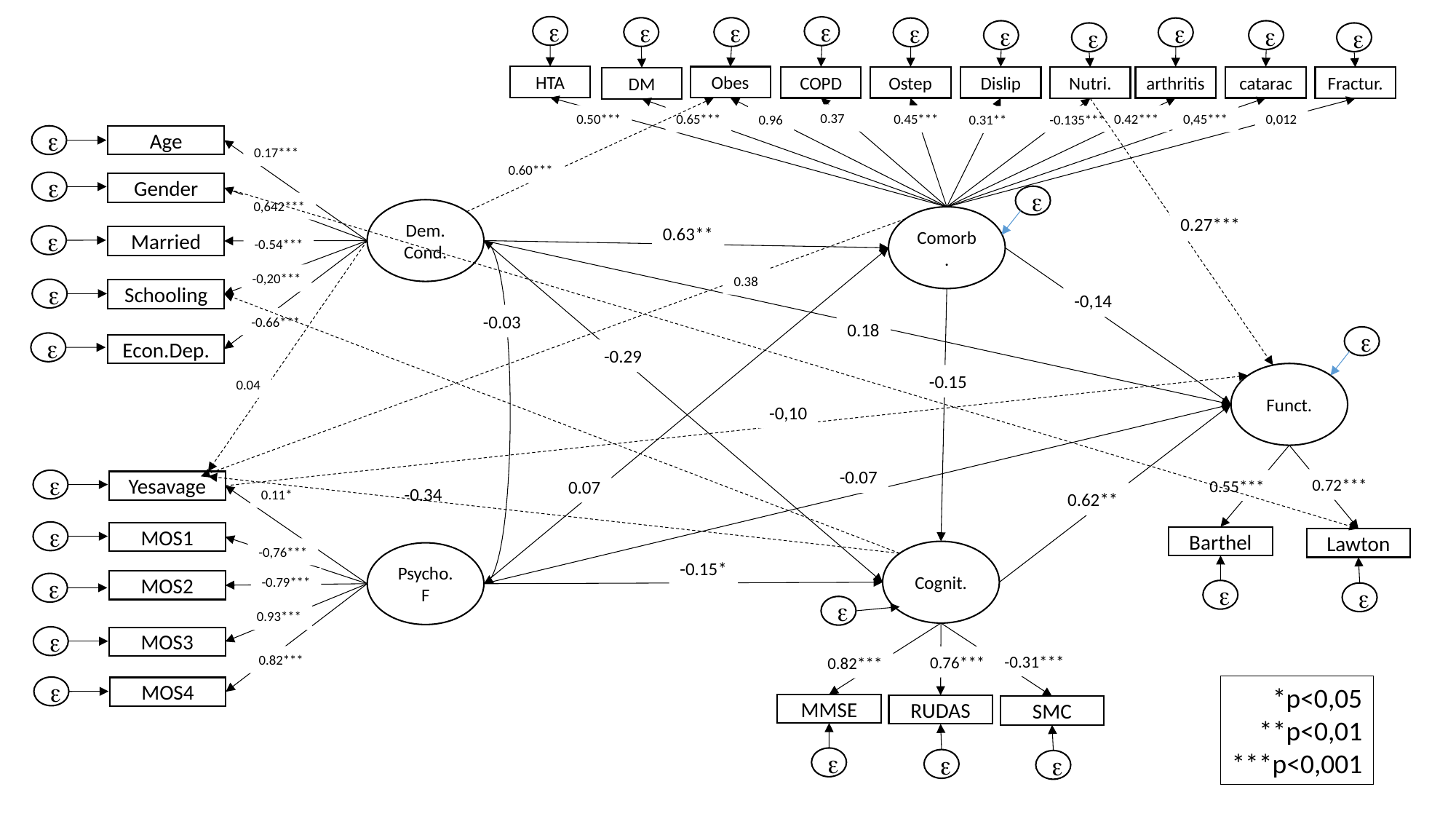

ε
ε
ε
ε
ε
ε
ε
ε
ε
ε
HTA
Obes
arthritis
catarac
Ostep
Dislip
COPD
Fractur.
Nutri.
DM
0.37
0.42***
0,45***
0.50***
0,012
0.65***
0.45***
0.96
-0.135***
0.31**
ε
Age
0.17***
0.60***
ε
Gender
ε
0,642***
Dem.
Cond.
Comorb.
0.27***
0.63**
ε
Married
-0.54***
-0,20***
0.38
ε
Schooling
-0,14
-0.03
-0.66***
0.18
ε
ε
Econ.Dep.
-0.29
Funct.
-0.15
0.04
-0,10
-0.07
0.72***
0.55***
0.07
ε
Yesavage
-0.34
0.11*
0.62**
ε
MOS1
Barthel
Lawton
-0,76***
Cognit.
Psycho.
F
-0.15*
-0.79***
MOS2
ε
ε
ε
ε
0.93***
ε
MOS3
-0.31***
0.82***
0.76***
0.82***
*p<0,05
**p<0,01
***p<0,001
ε
MOS4
MMSE
RUDAS
SMC
ε
ε
ε
